# Nudge for Good? Choice Defaults and Spillover Effects

**DOI:** 10.3389/fpsyg.2019.00178

**Published:** 2019-02-12

**Authors:** Claus Ghesla, Manuel Grieder, Jan Schmitz

**Affiliations:** Department of Humanities, Social and Political Sciences, Chair of Economics, Swiss Federal Institute of Technology (ETH) Zurich, Zurich, Switzerland

**Keywords:** defaults, nudge, licensing, consistency, spillovers

## Abstract

Policy makers increasingly use choice defaults to promote “good” causes by influencing socially relevant decisions in desirable ways, e.g., to increase pro-environmental choices or pro-social behavior in general. Such default nudges are remarkably successful when judged by their effects on the targeted behaviors in isolation. However, there is scant knowledge about possible spillover effects of pro-social behavior that was induced by defaults on subsequent related choices. Behavioral spillover effects could eliminate or even reverse the initially positive effects of choice defaults, and it is thus important to study their significance. We report results from a laboratory experiment exploring the subsequent behavioral consequences of pro-social choice defaults. Our results are promising: Pro-social behavior induced by choice defaults does not result in adverse spillover effects on later, subsequent behavior. This finding holds for both weak and strong choice defaults.

**JEL Classification:** C91, D01, D04

## 1. Introduction

Behavioral policy interventions from the toolkits of psychology and behavioral economics have gained increasing attention recently (e.g., List and Price, 2016; Liebe et al., 2018, for reviews of the literature). The goal of such interventions is to steer behavior in a desired direction when the use of classical policy instruments, such as taxes, subsidies, or command-and-control regulation, is not feasible and policies need to rely on the voluntary participation of actors (e.g., Croson and Treich, 2014; Kesternich et al., 2017).

One particularly prominent behavioral policy instrument are choice defaults. Policy makers (and other practitioners) make increasing use of choice defaults because they believe that defaults offer successful and cost-effective ways of triggering behavior change. Indeed, choice defaults appear to be very effective nudges for promoting “good” causes. For instance, defaults successfully promote pro-environmental choices such as the uptake of green energy contracts (Pichert and Katsikopoulos, 2008; Ebeling and Lotz, 2015), they strongly impact charitable donations (Altmann et al., 2014; Goswami and Urminsky, 2016), and they help increase retirement savings (Choi et al., 2003; Cronqvist and Thaler, 2004). Thus, even though there is a lively debate on the ethicality of using defaults as nudges (Bovens, 2009; Hausman and Welch, 2010; Desai, 2011; Sunstein, 2015), their distributional effects (Brown et al., 2011; Loefgren et al., 2012), and whether their use fits the criteria of “libertarian paternalism” (Carroll et al., 2009; Keller et al., 2011; Ghesla, 2017b), the effectiveness of default nudges for promoting “good” causes has generally been taken for granted.

However, for an accurate assessment of the overall effects of default nudges on a socially desired behavior, policy makers should take into account not only the direct impact of default nudges on targeted choices, but also potential spillover effects of the initial behavior triggered by the default on subsequent, related behaviors (see also d'Adda et al., 2017).^1^ In principle, such behavioral spillovers could amplify, eliminate or even reverse the initially positive effects of choice defaults, when judging their impact on the aggregate of relevant behaviors (for overviews see Truelove et al., 2014; Dolan and Galizzi, 2015). For instance, if nudging someone into a charitable donation crowds out other pro-social acts in the future, e.g., because of moral licensing (Khan and Dhar, 2006; Sachdeva et al., 2009; Mazar and Zhong, 2010), the net effect of the choice default for promoting pro-social behavior is clearly less positive—and could even become negative—than when no such spillover occurs.

In this paper, we use a laboratory experiment to study spillover effects of pro-social behavior triggered by choice defaults in a first stage on a *subsequent* pro-social behavior in a second stage. Our study is thus an intervention study of spillover effects (see Sintov et al., 2019), investigating whether default interventions can trigger behavioral spillovers to non-targeted, subsequent behavior. By doing so, our paper contributes to and links two strands of literature: on the one hand the literature studying behavioral spillovers (e.g., Meritt et al., 2010; Truelove et al., 2014; Dolan and Galizzi, 2015) and on the other hand the literature studying the effects of default nudges on pro-environmental or pro-social behavior (e.g., Thaler and Sunstein, 2003; Pichert and Katsikopoulos, 2008; Metcalfe and Dolan, 2012; Altmann et al., 2014; Sunstein and Reisch, 2014; Ebeling and Lotz, 2015; Goswami and Urminsky, 2016). To the best of our knowledge, there is only the study by d'Adda et al. (2017) that also links the literature on nudging interventions to the literature on behavioral spillovers and investigates the potential spillover effects of pro-social behavior triggered by nudges on subsequent behavior. D'Adda et al. (2017) use a similar design as ours in order to test relevant behavioral spillovers induced by various policy interventions, including a number of typical “nudges” such as choice defaults and information about social norms. They find that behavior influenced by traditional policy interventions in the form of monetary incentives or contractual regulation had positive spillover effects (mainly because of anchoring effects), whereas behavior triggered by nudging interventions had no spillover effects. However, with regard to choice defaults their results remained inconclusive, as their default manipulation did not produce a significant effect on the initial behavior. In our study, we ensured that the default manipulations yielded statistically significant effects on the targeted initial pro-social behavior. This allows testing the spillover effects of pro-social behavior triggered by successful default nudges on subsequent related decisions that were not directly targeted by the initial default nudge.

The existing empirical literature on behavioral spillovers in sequential pro-social decisions points to the possibility of moral licensing. After a first good deed, people can feel licensed to subsequently act in a negative way, thus resulting in negative spillovers of the initial positive behavior on the subsequent behavior (e.g., Monin and Miller, 2001; Jacobsen et al., 2010; Meritt et al., 2010; Conway and Peetz, 2012; Harding and Rapson, 2013; Tiefenbeck et al., 2013; Achtziger et al., 2015; Clot et al., 2016). As effective choice defaults in our setting increase pro-social behavior in the initial decision, they may trigger moral licensing tendencies leading people to compensate their high initial pro-social behavior (i.e., pro-social giving triggered by a default in our experiment) by subsequently less pro-social behavior. As such compensating behavior would undermine the overall effectiveness of default interventions, it is important to study whether pro-social behavior fostered through the use of pro-social default options leads to negative spillovers on subsequent, non-targeted pro-social behavior.

Contrary to moral licensing, the literature on behavioral spillovers also documents moral consistency effects according to which increased pro-social choices triggered by an intervention like a choice default should lead to even more pro-social behavior subsequently. However, many studies finding moral consistency did so in set-ups where the subsequent behavior was in the opposite domain than the initial behavior (i.e., pro-social behavior followed by anti-social behavior or vice versa, e.g., Freedman and Fraser, 1966; Beaman et al., 1983; Cialdini et al., 1995; Burger, 1999; Knez and Camerer, 2000; Fitzsimons and Shiv, 2001; Cherry et al., 2003; Grimm and Mengel, 2012; Baca-Motes et al., 2013; Brandon et al., 2017). As the goal of our study was to to investigate the behavioral spillover effects associated with choice defaults designed for fostering desirable, pro-social behavior, participants in our study faced an initial and a subsequent decision from the same domain (pro-social giving). This is different from making anti-social (e.g., cheating) decisions that harm others. Moreover, in set-ups with behavioral spillovers within the same (positive) domain (e.g., pro-environmental acts), positive spillovers are more likely when the conditions favor potential mediating mechanisms such as self-efficacy (as people learn that they are able and willing to perform certain behaviors, e.g., Steinhorst et al., 2015; Lauren et al., 2016), the cognitive accessibility of recent relevant behaviors (Sintov et al., 2019), or, relatedly, the self-signaling value of the behavior (Gneezy et al., 2012). By their nature, choice defaults do not seem likely to trigger these mediating pathways that could lead to positive spillovers, as defaults tend to affect behavior without people being explicitly aware of it (e.g., Smith et al., 2013), thus not fostering self-efficacy and making the pro-social behavior less easily cognitively accessible and thus also less relevant for self-signalling.

While previous literature thus suggests that moral licensing tendencies could be expected to occur if pro-social behavior is triggered by a choice default in a first decision, in our experiment we do not find that increased pro-social behavior triggered by choice defaults leads to negative spillovers on subsequent pro-social behavior that was not directly targeted by the default nudge. These results carry some positive messages for policy makers and choice architects. On the basis of our findings, there is currently no reason that choice architects need to worry about negative spillover effects from the use of pro-social choice defaults.

The remainder of this paper is organized as follows. Section 2 presents the experimental design. In section 3 behavioral hypotheses are presented. Section 4 summarizes the study results. Section 5 discusses relevant findings and concludes.

## 2. Experimental Design

To study whether choice defaults in a first *initial* decision affect behavior in an untreated *subsequent* decision we based our experimental design on a “sequential behavior paradigm,” which is typically used to study behavioral spillover effects experimentally (Mullen and Monin, 2016). For both decisions, we implemented dictator games (Kahneman et al., 1986; Forsythe et al., 1994) in order to have two very similar pro-social deeds as an instrument to uncover potential spillover effects of a default in one decision on a related subsequent decision without a default. The dictator game is a standard game in experimental economics and psychology with typically two players. One player is an active decision maker (she) who receives a certain monetary endowment, which she is free to divide between herself and another (passive) player, the recipient (he). The recipient can be another person, but he can also be an environmental or social cause or charity to which decision makers can donate to. Importantly, the recipient cannot influence how much the decision maker decides to transfer and he has no way of rejecting the transfer. The game thus serves as a measure of voluntary pro-social behavior by the decision maker. It has been extensively used in pro-social decision research (see Engel, 2011, for a meta-analysis).

Specifically, in our study, in the first decision participants played a dictator game paired with a charity as the recipient (“Dictator Stage I”). In the subsequent second decision, participants played another dictator game in which they were paired with a randomly allotted person in the same laboratory session (“Dictator Stage II”). In both stages, participants could be either selfish (and keep the money for themselves) or pro-social (and share some of their endowment with the recipient). Importantly, if there are spillover effects, the decision in Dictator Stage II may depend on the behavior in Dictator Stage I and on the presence and strength of a choice default in that stage.

### 2.1. Method and Procedures

#### 2.1.1. Dictator Stage I

Participants played a dictator game paired with a recipient in form of a charitable organization. They could choose from nine different charities, which served a well-balanced set of purposes, such as charities that deal with environmental and nature conservation, human rights, or health related matters. Thus, we tried to preclude situations in which participants would have liked to donate, but could not find a suitable charity to do so (Crumpler and Grossman, 2008). Participants received information on each charity by reading a statement of purpose.^2^

Participants received information about each charity, which they had to read before they were able to make a choice.^3^ Once they had read about all charities, participants decided to which of the nine charities (only one could be selected) and how much to give. Participants received a total amount of 200 experimental points (ECU) for their choice, of which they kept 100 points as a show-up fee. 100 ECU remained to decide on how much to donate to a charity. Participants also had the option to donate nothing and keep all experimental points for themselves.

We implemented three treatment variations in Dictator Stage I:

1. **NO DEFAULT**: Participants could choose actively if and how much to donate to a charity. They had to actively type the desired amount into an input box. The input box was initially blank.
2. **WEAK DEFAULT**: We nudged participants into being fully pro-social and donating the maximum possible amount to a charity by default. The default donation was thus pre-set to the maximum amount participants could donate (100 ECU). Participants could change the pre-set amount simply by clicking on a box and entering the desired donation.
3. **STRONG DEFAULT**: We again nudged participants into being fully pro-social by setting the default donation to the maximum possible amount that could be donated. In order to change the amount, participants first had to perform a slider task (Gill and Prowse, 2018). Specifically, to change the default donation, participants had to shift 48 sliders to a value of 50. Only after having completed the task, participants could change the donation amount. If they did not complete the slider task, they had to donate the default amount.

Many defaults used in charitable giving (Altmann et al., 2014) or pro-environmental settings (see Brown et al., 2013; Egebark and Ekstroem, 2016) are comparable to our weak default treatment. However, the literature provides multiple explanations for why people stick to defaults. For example, defaults may be set such that it may be rational to follow the default (Croson and Treich, 2014), they may convey information about certain choices over others and signal quality (Dinner et al., 2011; Coffman et al., 2015), or following the default may simply be cognitively less challenging (Sintov and Schultz, 2017). The latter point indicates that often, defaults seem to work (i.e., people stay with the default) because it is laborious for people to make an active choice and to opt out of the default. Our strong default treatment thus varies the cost of opting out. Taken together, our two default treatments accommodate the fact that opting-out of the default may be more or less complex in different situations.

We completed the experimental design with a two-tiered control strategy:

1. **CONTROL INCOME**: Participants did not participate actively in Dictator Stage I, but received lump-sum payments in addition to their show-up fees. The amounts of these lump-sum payments were derived from the distributions of donation amounts participants chose in the treatment conditions outlined above. Thus, each donation decision in the NO DEFAULT, WEAK DEFAULT, and STRONG DEFAULT treatments was matched with a lump-sum payment a participant received in the CONTROL INCOME condition. In purely monetary terms, participants in CONTROL INCOME thus arrived at Dictator Stage II in exactly the same situation as a matched participant from one of the treatments, however without having made a donation decision in Dictator Stage I. Eliminating Dictator Stage I behavior while controlling for any possible income effects provides us with a conservative baseline to which we can compare the Dictator Stage II decisions in our three main treatments.
2. **CONTROL PASSIVE GIVING**: Participants received the identical lump-sum payments according to the same procedure as participants in CONTROL INCOME. Yet, they did participate (to a limited extent) in Dictator Stage I by choosing the charity to which a pre-defined donation was made. By letting participants choose the charity to which the donation was administered, we made sure that the altruistic utility component, i.e., the individual knowledge that there had been a donation in Dictator Stage I was comparable to participants' utility in the NO DEFAULT, WEAK DEFAULT, and STRONG DEFAULT treatments.^4^ Additionally, as participants read about the charities in Dictator Stage I in the treatment condition, this condition also controls for any possible priming effects of that task on the subsequent decision in Dictator Stage II.

#### 2.1.2. Dictator Stage II

Participants played a standard dictator game with another participant as the recipient. Each participant was thus paired randomly with another participant in the same session. Both participants remained completely anonymous with respect to each other and were not able to influence the other participant's decision. To maximize the number of observations, we used a variant of the strategy method (Selten, 1967) and elicited choices for both roles of the dictator and the recipient respectively. The strategy method is a common experimental procedure to elicit all possible choices in a behavioral game from one participant (see Brandts and Charness, 2011, for a more detailed discussion and for evidence that treatment effects found in direct response experiments also replicate with the strategy method). In our setting this meant that we asked participants to make decisions for both roles that exist in the game, the dictator (i.e., how much of their endowment would they like to share with the recipient) and the recipient. Each participant thus decided on the allocation of 200 experimental points between herself and the paired recipient. However, it was common knowledge that only one decision of each pair of participants was going to be implemented, and that the computer would randomly determine which one. Dictator Stage II was completely identical for participants in all treatments and control conditions. The decisions made in this stage constitute our main dependent variable. Table 1 summarizes the experimental parameters.

**Table 1:** Overview of experimental parameters.

	**Dictator Stage I**		**Dictator Stage II**
	**Show-up fee**	**ECU for decision**	**ECU for decision**
T1 NO DEFAULT	100	100	200
T2 WEAK DEFAULT	100	100	200
T3 STRONG DEFAULT	100	100	200
C1 CONTROL INCOME	100+$\hat{X}$	–	200
C2 CONTROL PASSIVE GIVING	100+$\hat{X}$	Fixed: (100-$\hat{X}$)	200

*Participants in CONTROL INCOME and CONTROL PASSIVE GIVING received a lump-sum payment $\hat{X}$ matching the distribution of the donated amounts in Dictator Stage I in the treatment conditions (see Appendix B in Supplementary Material for details on the matching procedure). In Dictator Stage II, each participant decided on the allocation of 200 ECU, however, only one decision within each participant pair was implemented. 100 ECU ≡ CHF 10*.

#### 2.1.3. Participants and Procedures

We conducted 23 sessions with a total of 678 participants at the Decision Science Laboratory (DeSciL) at ETH Zurich. The recruitment process followed standard protocols at the laboratory and we did not apply any exclusion rules, e.g., based on study or subject level. We recruited participants using hroot, a software tool frequently used to recruit participants for behavioral economics experiments and that allows for randomized invitation to experimental sessions (see Bock et al., 2014). The participant pool consisted of students at the University of Zurich and the Swiss Federal Institute of Technology (ETH) in Zurich. In our final sample, 53% of participants were women and the mean age was 22.9 years. Table A1 in Appendix A (Supplementary Material) provides further descriptive statistics on the participant sample (including, in addition to age and gender, measures for income, education, Big 5 traits, need for cognition, reactance, regret, and IQ for each of the experimental conditions as well as in the sample overall.)

**Figure 1:**
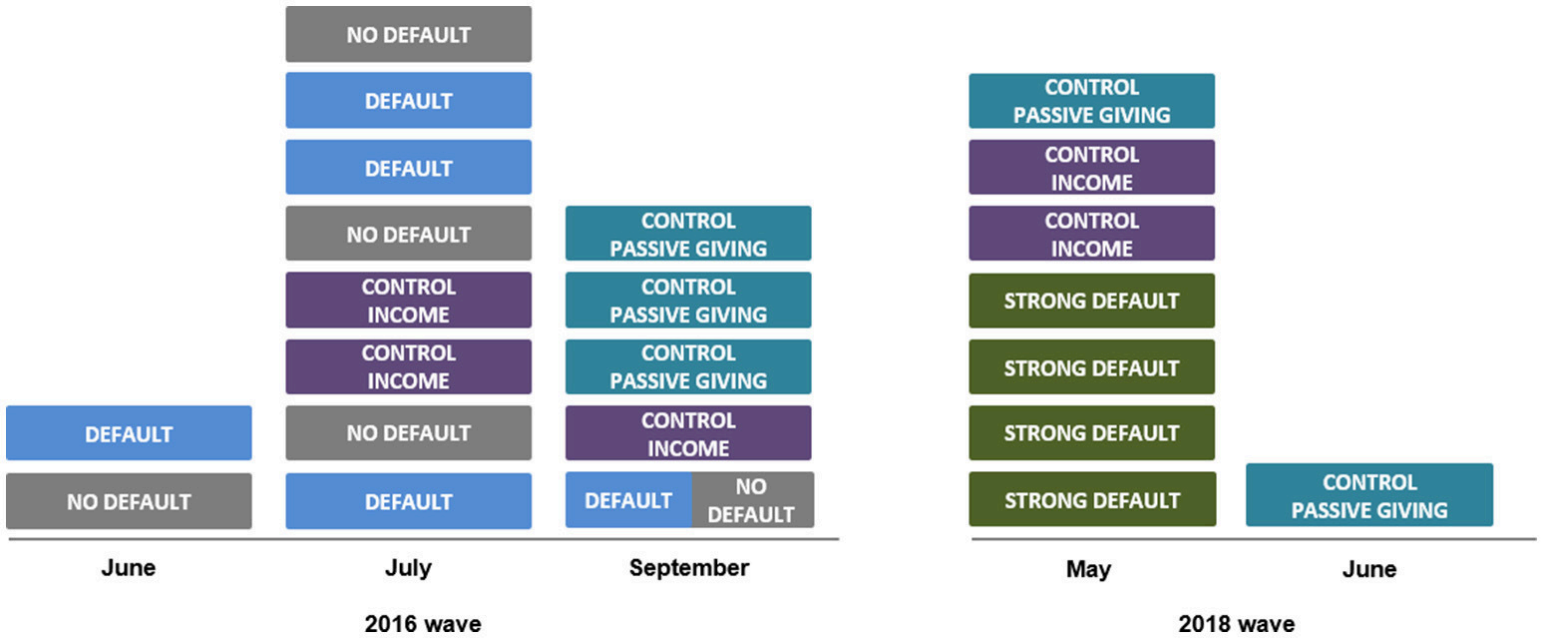
The figure illustrates the timeline of our data collection. Each box represents one experimental session (lasting for around 50 min each), the label indicates the experimental condition implemented in that session and, in parentheses, we indicate the number of participants in the session. The split box at the very bottom for September 2016 indicates the one treatment session that we conducted in a within fashion in order to balance cell-sizes because of no-shows in previous sessions (see Footnote 5).

We collected data for the NO DEFAULT, WEAK DEFAULT and the corresponding control conditions in June, July and September 2016. The data for the STRONG DEFAULT and its corresponding control conditions were collected in May and June 2018. It is possible that unobserved changes in the participant pool between 2016 and 2018 could have affected participants' behavior. However, when we compare the 2016 and the 2018 data of the corresponding control conditions (CONTROL INCOME and CONTROL PASSIVE GIVING), we do not find any significant differences in behavior (*p*>.100 for all comparisons), which is why we pool the data from 2016 and 2018 for the analyses. Figure 1 provides an illustration of the data collection timeline. Each box in the figure represents an experimental session and displays the experimental condition(s) implemented in that session.

In order to obtain the amounts and the distribution of the lump-sum payments ($\hat{X}$) in the control groups, we ran four sessions of NO DEFAULT and WEAK DEFAULT first (in the 2016 wave). Subsequently, we varied treatments and control between sessions^5^ and sessions were executed such that treatments and controls were evenly distributed across different times and days. We followed the same procedure for the STRONG DEFAULT treatment and the corresponding CONTROL INCOME and CONTROL PASSIVE GIVING conditions in the data collection wave in 2018. Thus, we first conducted four sessions in the STRONG DEFAULT treatment to gather information about giving in Dictator Stage I and the income distribution for Dictator Stage II. We computerized the experiment using z-tree, a software tool frequently used in experimental economics that allows conducting anonymous interactive decision making experiments in the laboratory (see Fischbacher, 2007). An experimental session lasted roughly 50 minutes.

At the beginning of a session, participants were randomly assigned to computer-equipped cubicles. Common rules for participation were read aloud and participants signed a consent form. They received on-screen instructions for each part of the study (see Appendix C in Supplementary Material that contains the entire set of experimental instructions provided to participants). Participants knew that the study would consist of several parts, but the contents of each part were not revealed before the respective instructions were provided. In order to ensure comprehension, participants had to answer control questions before each part. When participants had comprehension questions, the experimenter answered individually and in private.

Participants first completed Dictator Stage I (except in CONTROL INCOME). Subsequently, we included a filler task between Dictator Stage I and II. In this task, participants completed a shortened version of an IQ-test after Catell (1940). The test was divided into two parts, each part lasting for exactly 90 seconds. The intention of the filler task was to temporally separate Dictator Stage I and II. This separation may be of importance when reviewing the proposed underlying psychological mechanisms of consistency or licensing effects. One line of research argues that individuals store moral credits when behaving “good,” which they then use later on, for instance, to offset a subsequent behavior (Jordan et al., 2011). Another line of research states that individuals use initial “good” behavior as a credential to interpret negative subsequent behavior as non-negative (Monin and Miller, 2001). The filler task serves both mechanisms as, on the one hand, it provided sufficient time for participants to build up moral credits, and on the other hand, it was still short enough so that in the subsequent behavior participants would remember their initial behavior. Additionally, the filler task limits the potential for demand (Zizzo, 2010) and anchoring effects (e.g., d'Adda et al., 2017) and adds to the external validity of the results, as in relevant real-life settings an initial behavior is most likely not followed immediately by a relevant subsequent behavior. After the filler task, participants proceeded to Dictator Stage II. Upon completion of these tasks, they received feedback on their final payoff and were asked to fill in a supplemental questionnaire. The average payment was approximately CHF 26. Moreover, participants donated CHF 2,155 to the nine different charities.

## 3. Behavioral Predictions and Hypotheses

The experiment was designed to study potential behavioral spillover effects arising from initial pro-social giving behavior on subsequent giving behavior in a related decision. Particularly, we were interested in testing whether the use of choice defaults that triggered giving in the initial behavior in Dictator Stage I would affect behavioral spillovers to the subsequent decision in Dictator Stage II.

To guide our analysis in section 4, we provide behavioral predictions and testable hypotheses grounded in existing literature in this section. Because we want to test the effect of behavioral spillovers following pro-social behavior in conditions with a pre-set default, we first present hypotheses about Dictator Stage I giving behavior in the differently strong default treatments in section 3.1. Further, we present hypotheses about potential spillover effects arising from giving in Dictator Stage I on giving behavior in Dictator Stage II in section 3.2.

### 3.1. The Effect of Defaults on Giving in Dictator Stage I

A large body of literature documents that when presented with choice defaults, individuals oftentimes follow the pre-set option (e.g., Thaler and Sunstein, 2003; Altmann et al., 2014; Ebeling and Lotz, 2015). As we are interested in identifying potential spillover effects of pro-social behavior induced by choice defaults on subsequent, non-targeted pro-social behavior, providing further evidence for the direct effects of choice defaults is not the main concern of our study. However, to be able to study potential spillover effects, we first need to establish the presence of a default effect in our study on the directly targeted pro-social behavior (giving in Dictator Stage I). Specifically, we use two different defaults in Dictator Stage I. The defaults differ in the effort level required to change the pre-set donation amount. While reasons to follow default decisions are diverse, the literature also indicates that effort is a prime factor preventing individuals to change pre-set choices (Brown et al., 2013; Altmann et al., 2014; Egebark and Ekstroem, 2016; Sintov and Schultz, 2017). Based on the existing literature on choice defaults, we thus present Hypotheses 1a-c:

**Hypothesis 1:** The effect of defaults on giving in Dictator Stage I

H1a The weak default nudge increases giving in Dictator Stage I compared to giving in the no default condition.
H1b The strong default nudge increases giving in Dictator Stage I compared to giving in the no default condition.
H1c The strong default nudge increases giving in Dictator Stage I compared to the weak default nudge.

Note that a non-rejection of H1a and H1b is indispensable to study our main research question which concerns the impact of choice defaults on potential spillover effects of first on second stage behavior (see Hypothesis 2 below). Thus, without the significant effects of defaults on giving in Dictator Stage I, an analysis of possible spillover effects on Dictator Stage II is obsolete.

### 3.2. Spillover Effects Arising From Giving in Dictator Stage I

Hypothesis 1 thus merely represents a necessary condition to investigate spillover effects from default induced giving in Dictator Stage I on giving in Dictator Stage II. Behavioral spillover effects in decision settings without choice defaults have been widely studied and the related literature on behavioral spillover effects from identical and closely related pro-social decisions points to the importance of moral licensing (e.g., Schmitz, forthcoming; Tiefenbeck et al., 2013; Hofmann et al., 2014; Achtziger et al., 2015; Effron and Conway, 2015; Sass et al., 2015). Individuals who give to others (or to charity) in a first decision tend to show less of this behavior in subsequent giving decisions. Since we use two related consecutive pro-social decisions it is likely to observe negative behavioral spillovers in our setting too. Following the arguments presented in the literature, higher giving induced by the default in Dictator Stage I should lead to negative spillover effects on giving in Dictator Stage II. We present Hypotheses 2a-c:

**Hypothesis 2:** The spillover effects of charitable giving in default conditions in Dictator Stage I on giving in Dictator Stage II

H2a Compared to the no default condition, the higher initial giving to charity induced by the weak choice default in Dictator Stage I leads to lower giving in Dictator Stage II.
H2b Compared to the no default condition, the higher initial giving to charity induced by the strong choice default in Dictator Stage I leads to lower giving in Dictator Stage II.
H2c Compared to the weak default condition, the higher initial giving to charity induced by the strong choice default in Dictator Stage I leads to lower giving in Dictator Stage II.

These moral licensing hypotheses stand in contrast to literature describing moral consistency effects, i.e., higher pro-social behavior following anti-social behavior in an initial decision (e.g., Freedman and Fraser, 1966; Beaman et al., 1983; Cialdini et al., 1995; Burger, 1999; Knez and Camerer, 2000; Fitzsimons and Shiv, 2001; Cherry et al., 2003; Grimm and Mengel, 2012; Baca-Motes et al., 2013; Brandon et al., 2017). As discussed in the introduction, however, this literature identifies spillover effects from a first decision on a second decision where the first decision is conceptually different from the second. In our study, both decisions involve giving to others, and are thus highly similar. Moreover, as also discussed in the introduction, choice defaults seem unlikely to favor mediating mechanisms for positive spillovers such as self-efficacy (Steinhorst et al., 2015; Lauren et al., 2016), cognitive accessibility (Sintov et al., 2019) or self-signaling (Gneezy et al., 2012).

## 4. Results

In presenting our results, we follow the structure of the hypotheses laid out in section 3 by first testing whether our default manipulations had a significant effect on giving in Dictator Stage I (Hypothesis 1) and then testing whether the choice defaults affected the spillover of giving in Dictator Stage I on giving in Dictator Stage II (Hypothesis 2). Finally, we contrast the findings in the default treatments with behavior in the different control conditions disentangling possible income effects and altruistic motives from spillover effects arising from giving in Dictator Stage I. A final regression analysis provides a comprehensive overview of all the results that are concerned with potential spillover effects.

As a first descriptive analysis, Table 2 provides an overview of giving choices [in experimental points (ECU)] in Dictator Stage I and II for all treatment and control conditions.^6^

**Table 2:** Summary statistics.

		**Giving (ECU)**	
**Treatments**	**N**	**Dictator Stage I**	**Dictator Stage II**
NO DEFAULT	129	27.44 (25.38)	35.89 (36.80)
WEAK DEFAULT	129	34.26 (31.47)	39.69 (39.80)
STRONG DEFAULT	128	58.98 (43.82)	40.94 (43.15)
**Control Conditions**	**N**		**Dictator Stage II**
CONTROL INCOME (NO DEFAULT matching)	49	–	39.39 (44.32)
CONTROL INCOME (WEAK DEFAULT matching)	49	–	40.20 (40.59)
CONTROL INCOME (STRONG DEFAULT matching)	50	–	50.80 (42.71)
CONTROL PASSIVE GIVING (NO DEFAULT matching)	46	–	34.57 (39.87)
CONTROL PASSIVE GIVING (WEAK DEFAULT matching)	46	–	43.70 (39.80)
CONTROL PASSIVE GIVING (STRONG DEFAULT matching)	52	–	43.65 (40.44)

*Giving is denoted in ECU. Standard deviations are in parentheses. The data for the six control conditions are split into the respective income matching category, i.e., NO DEFAULT, WEAK DEFAULT, STRONG DEFAULT*.

### 4.1. Effects of Choice Defaults on Targeted Behavior

#### 4.1.1. The Effect of a Weak Default on Giving in Dictator Stage I

Our weak default manipulation in Dictator Stage I had a significant effect on donation levels. Participants in the WEAK DEFAULT treatment donated on average 25% more than participants in the NO DEFAULT condition (34.26 ECU vs. 27.44 ECU). Thus, in line with H1a, the pro-socially set weak default marginally increased overall giving [*t*_(256)_ = −1.92, *p* = 0.056, Cohen's *d* = 0.24)].^7^ Furthermore, participants in the WEAK DEFAULT treatment also had a marginally significant higher prevalence of choosing exactly the pro-socially set default amount (= 100 ECU) (11.6% in WEAK DEFAULT vs. 4.6% in NO DEFAULT, *z* = 3.32, *p* = 0.069, *n*_1_ = 129, *n*_2_ = 129).

The default effect can be further partitioned when considering giving as a two-stage decision process. Participants first decide whether they want to donate or not. Once chosen to donate, they decide on the size of their gift (e.g., Moffatt, 2016, who deems such an analysis particularly important for Dictator Game data). Our default manipulation did not affect the number of participants who decided to give nothing (24.8% in WEAK DEFAULT vs. 24.8% in NO DEFAULT, *z* = 0.00, *p* = 1.000, *n*_1_ = 129, *n*_2_ = 129). However, it did affect donation levels once participants decided to give. Comparing only participants who decided to give a positive amount, the effect of the weak default holds. Donations in the WEAK DEFAULT treatment (45.57 ECU) are on average 25% higher than in the NO DEFAULT treatment (36.49 ECU). This difference of 9.08 ECU is statistically significant [*t*_(192)_ = −2.45, *p* = 0.015].

#### 4.1.2. The Effect of a Strong Default on Giving in Dictator Stage I

In line with H1b, participants in the STRONG DEFAULT treatment gave on average 114% more to charity than participants in the NO DEFAULT treatment (58.99 ECU vs. 27.44 ECU). Moreover, and in line with H1c, in Dictator Stage I, participants in the STRONG DEFAULT treatment donated on average 72% more to charity than participants in the WEAK DEFAULT treatment (58.98 ECU vs. 34.26 ECU). Therefore, supporting H1b and H1c our stronger default manipulation significantly increased donation levels when compared to these two conditions [STRONG DEFAULT vs. NO DEFAULT *t*_(255)_ = −7.07, *p* < 0.001, Cohen's *d* = 0.77; STRONG DEFAULT vs. WEAK DEFAULT: *t*_(255)_ = −5.20, *p* < 0.001, Cohen's *d* = 0.54]. Furthermore, participants in the STRONG DEFAULT treatment were also more likely to donate exactly the pre-set default amount when compared to participants in the WEAK DEFAULT treatment and when compared to participants in the NO DEFAULT treatment (proportion tests: 49.6% in STRONG DEFAULT vs. 4.6% NO DEFAULT: *z* = 63.70, *p* < 0.001, *n*_1_ = 128, *n*_2_ = 129; 49.6% in STRONG DEFAULT vs. 11.6% in WEAK DEFAULT: *z* = 42.04, *p* < 0.001, *n*_1_ = 128, *n*_2_ = 129). However, our strong default manipulation did not affect the number of participants who decided to give nothing (22.65% in STRONG DEFAULT vs. 24.8% in NO DEFAULT: *z* = 0.07, *p* = 0.796, *n*_1_ = 129, *n*_2_ = 129; 22.65% in STRONG DEFAULT vs. 24.8% WEAK DEFAULT: *z* = 0.07, *p* = 0.796, *n*_1_ = 129, *n*_2_ = 129).

Nevertheless, the strong default did affect donation levels once participants decided to give a positive amount. Participants who gave a positive amount to charity donated on average 67% more in STRONG DEFAULT (76.26 ECU) compared with participants in the WEAK DEFAULT treatment (45.57 ECU). This difference of 30.69 ECU is statistically significant [*t*_(194)_ = −6.86, *p* < 0.001]. Further, participants in the STRONG DEFAULT treatment (ECU 76.26) gave on average 109% more than participants in NO DEFAULT treatment (36.49 ECU). This difference of 39.77 ECU is again statistically significant [*t*_(194)_ = −9.58, *p* < 0.001].

### 4.2. Spillover Effects

#### 4.2.1. The Spillover Effect of Giving in the Weak Default Treatment in Dictator Stage I on Giving in Dictator Stage II

In order to assess the spillover effect from giving in a weak default regime in stage one to giving behavior in stage two (H2a), we compare giving in Dictator Stage II between the WEAK DEFAULT and NO DEFAULT treatments. Table 2 reveals that participants in both treatments gave about one fifth of their endowment to the paired recipient. In the NO DEFAULT treatment, participants gave 35.89 ECU (18% of their endowment). In the WEAK DEFAULT treatment, average giving amounted to 39.69 ECU (20% of the endowment). The difference of less than 4 ECU is not statistically significant [*t*_(256)_ = −0.80, *p* = 0.427, Cohen's *d* = 0.10]. There is thus no support for H2a, as we do not find a significant spillover effect in the weak default treatment. We summarize this finding as our first result:

**Result 1**. *There are no behavioral spillover effects from giving in stage one in the WEAK DEFAULT treatment on subsequent giving. Higher initial giving in Dictator Stage I in the WEAK DEFAULT treatment does not lead to lower giving in Dictator Stage II compared with the NO DEFAULT treatment*.

### 4.3. The Effect of Giving in the Strong Default Treatment in Dictator Stage I on Giving in Dictator Stage II

Table 2 documents that participants in the STRONG DEFAULT treatment also gave about one fifth of their endowment to the other recipient. This is very similar to the amounts given by participants in the WEAK DEFAULT treatment and the NO DEFAULT treatment. In fact, there are no differences in Dictator Stage II giving between treatments that are statistically significant [WEAK DEFAULT vs. STRONG DEFAULT: *t*_(255)_ = −0.24, *p* = 0.810, Cohen's *d* = 0.03; NO DEFAULT vs. STRONG DEFAULT *t*_(255)_ = −1.01, *p* = 0.314, Cohen's *d* = 0.13], and there is thus no support for either H2b or H2c. It does not seem to be the case that choice defaults on giving in Dictator Stage I lead to moral licensing in Dictator Stage II. We summarize these findings in our second result:

**Result 2**. *There are no behavioral spillover effects from giving in stage one in the STRONG DEFAULT treatment on subsequent giving. Higher initial giving in Dictator Stage I in the STRONG DEFAULT treatment does not lead to lower giving in Dictator Stage II compared with the NO DEFAULT treatment*.

**Figure 2:**
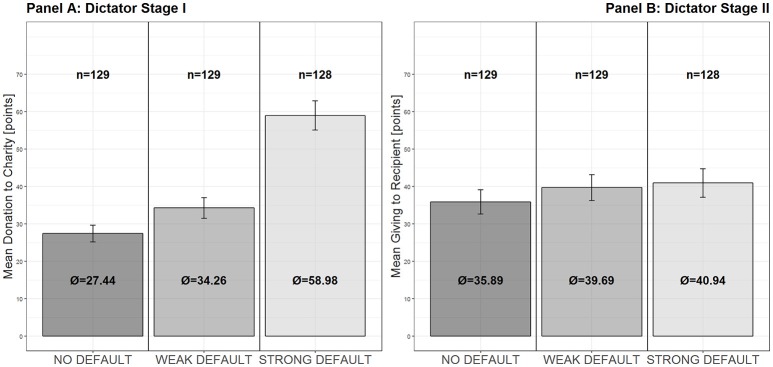
Choices in Dictator Stage I and II. Panel **(A)** Shows giving decisions (mean points donated to charities) in Dictator Stage I for NO DEFAULT, WEAK DEFAULT and STRONG DEFAULT. Panel **(B)** Shows mean giving (points given to recipient) in the Dictator Stage II for the three treatment conditions. Error-bars denote plus/minus one standard error of the mean.

Figure 2 illustrates the findings presented so-far. Figure 2A of the figure illustrates the statistically significant impact of both the weak and the strong default on giving in Dictator Stage I (with the STRONG DEFAULT condition adding a significant increase to donation levels compared to the WEAK DEFAULT). Figure 2B of the figure shows that in the untreated Dictator Stage II no differential spillover of the initial decision can be observed, as we do not find significant differences between the experimental conditions.

### 4.4. Income and Altruistic Motivations

To put our results concerning potential spillover effects to a more conservative test and to ensure the robustness of our findings, we employed a two-tiered control strategy. Solely comparing choices in the NO DEFAULT treatment with choices in the WEAK DEFAULT treatment and the STRONG DEFAULT treatment in Dictator Stage II may omit relevant differences between the treatments related to income effects and altruistic motivations. Specifically, because of their donation decision, participants arrived with different amounts of money in Dictator Stage II in the default treatments compared with participants in the NO DEFAULT treatment. This, on the one hand, impacts income of participants in the default treatments. On the other hand, motivations of altruism may also be affected by the higher donations in Dictator Stage I in the default treatments. To control for pure income effects, we employ the CONTROL INCOME condition in which participants did not make a donation decision in Dictator Stage I but had the same income as participants in the default treatments when they made their decisions in Dictator Stage II. To control also for altruistic motivations, we conducted the CONTROL PASSIVE GIVING condition in which participants also had the same income as participants in the default treatments in Dictator Stage I, but without having made an active donation in Stage I and instead simply learning that a donation was made to a charity (and in which amount) to keep altruistic utility constant. We compare giving in Dictator Stage II in these conditions to giving in in Dictator Stage II in the NO DEFAULT treatment and the WEAK DEFAULT and STRONG DEFAULT treatment respectively.

The results from our control conditions further support Results 1 and 2. Participants' choices in the NO DEFAULT treatment and the WEAK DEFAULT treatment were not significantly different to those of the matched cases in the CONTROL INCOME condition and the CONTROL PASSIVE GIVING condition [NO DEFAULT (35.89 ECU) vs. CONTROL INCOME (39.39 ECU): *t*_(176)_ = −0.53, *p* = 0.594; NO DEFAULT (35.89 ECU) vs. CONTROL PASSIVE GIVING (34.57 ECU): *t*_(173)_ = 0.21, *p* = 0.838; WEAK DEFAULT (39.69 ECU) vs. CONTROL INCOME (40.20 ECU): *t*_(176)_ = 0.08, *p* = 0.939; WEAK DEFAULT (39.69 ECU) vs. CONTROL PASSIVE GIVING (43.70 ECU): *t*_(173)_ = 0.57, *p* = 0.567]. Similarly, supporting Result 2, participants' choices in the STRONG DEFAULT treatment were not significantly different to those in the CONTROL INCOME condition or the CONTROL PASSIVE GIVING condition [STRONG DEFAULT (40.94 ECU) vs. CONTROL INCOME (50.8 ECU): *t*_(176)_ = 1.37, *p* = 0.171; STRONG DEFAULT (40.94 ECU) vs. CONTROL PASSIVE GIVING (43.65 ECU): *t*_(178)_ = 0.39, *p* = 0.697].^8^

Thus, putting potential spillover-effects to a more rigorous test by controlling for altruistic motivations and income effects reinforces our Results 1 and 2. Neither different incomes nor different altruistic motivations resulting from higher giving in Dictator Stage I seem to impact giving in Dictator Stage II.

As a final step, in Table 3 we report the results from regression analyses allowing to analyze whether spillover effects differed between the experimental conditions when controlling for potential income effects at the individual level. Note that for the pairwise comparisons of the default treatments to the CONTROL INCOME and the CONTROL PASSIVE GIVING conditions based on *t*-tests reported above, we had to split the observations from the CONTROL INCOME and CONTROL PASSIVE GIVING conditions into groups matching the respective treatment conditions (see Appendix B in Supplementary Material for details). The splitting into groups was conducted randomly, but it reduces statistical power. The regression approach avoids this splitting and has the advantage that instead we can simply add the individual monetary income a participant had received in the experiment up to Dictator Stage II as a control variable. This increases statistical power and thus provides an even stronger test of the findings we have established in section 4.2.

**Table 3:** Regression models: giving in dictator stage II.

**DV: Giving to Recipient**	**OLS**	**LPM**	**gamma-GLM**
		**Two-part model**	
Intercept	40.806^***^	0.738^***^	4.008^***^
	(3.742)	(0.043)	(0.066)
WEAK DEFAULT	2.379	−0.042	0.085
	(5.051)	(0.057)	(0.091)
STRONG DEFAULT	−4.361	−0.199^***^	0.215^**^
	(5.413)	(0.062)	(0.104)
CONTROL INCOME	2.709	−0.069	0.168^**^
	(5.150)	(0.058)	(0.087)
CONTROL PASSIVE GIVING	−0.051	−0.065	0.096
	(5.091)	(0.059)	(0.090)
Income before DG II	−13.600^***^	−0.218^***^	−0.052
	(4.972)	(0.059)	(0.102)
WEAK DEFAULT × Income before DG II	−6.239	−0.036	−0.067
	(6.376)	(0.075)	(0.126)
STRONG DEFAULT × Income before DG II	4.523	0.074	0.070
	(5.819)	(0.069)	(0.120)
CONTROL INCOME × Income before DG II	13.736^**^	0.240^***^	0.023
	(6.119)	(0.071)	(0.114)
CONTROL PASSIVE GIVING × Income before DG II	13.342^**^	0.196^***^	0.074
	(6.079)	(0.071)	(0.118)
Observations	678	678	443
*R*^2^	0.059	0.085	–
F(9, 668) / F(9, 668)/ χ^2^(9)	4.706	6.899	7.299

*+p < 0.10*;

* p < 0.05;

** *p < 0.01*;

*** *p < 0.0001*.

*Robust standard errors are in parentheses. The dependent variable is giving to the recipient in Dictator Stage II. NO DEFAULT is the omitted treatment captured by the intercepts. “Income before DG II” represents the (mean-centered) monetary income a participant had earned in the experiment when arriving at Dictator Stage II (partly endogenously determined in NO DEFAULT, WEAK DEFAULT, and STRONG DEFAULT, exogenously assigned in control treatments). Gamma-GLM estimates are on a log-scale. The two-part model fits the data better than the OLS specification subsuming the complete data. The combined log-likelihood of the two-part model is –2628.207 compared to –3454.219 of the OLS. The table was compiled using the “stargazer” tool by Hlavac (2018)*.

In the regressions reported in Table 3, the variable “Income before DG II” captures the monetary income a participant had earned in the experiment before making the giving decision in Dictator Stage II. We include dummies for our experimental conditions, with the NO DEFAULT treatment being the omitted base category. We interact the dummies for the experimental conditions with the “Income before DG II” variable to allow for the likely possibility that the effects of this variable are different between the experimental conditions. The reason is that the “income” with which a participant arrived in Dictator Stage II was endogenously determined through participants' giving in the NO DEFAULT, WEAK DEFAULT, and STRONG DEFAULT treatments, whereas it was exogenously assigned through the matching procedure in the CONTROL INCOME and CONTROL PASSIVE GIVING conditions.^9^

The treatment dummies in the regressions reported in Table 3 can be interpreted straightforwardly as capturing a difference in giving in Dictator Stage II between the respective treatment and the omitted base category, the NO DEFAULT condition, while controlling for income effects. The non-significant coefficients for the treatment dummies for WEAK DEFAULT and STRONG DEFAULT in the OLS regression thus indicate that, on average and compared to the NO DEFAULT treatment, neither a weak nor a strong default in the initial donation decision in Dictator Stage I led to different giving decisions in Dictator Stage II. Thus, despite the defaults significantly affecting the giving decisions in Dictator Stage I, there was no spillover effect of this increased giving in Dictator Stage I on Dictator Stage II. There were also no significant differences according to the OLS regression when comparing WEAK DEFAULT and STRONG DEFAULT to the two control conditions and WEAK DEFAULT and STRONG DEFAULT with each other (*p* > .100 for all post-estimation F-tests for these comparisons). The low *R*^2^ values correspond to this lack of statistically significant differences between the experimental treatments.

Additionally, we again analyze the data on giving decisions in Dictator Stage II as a two-step decision process. This analysis is based on the assumption that participants first decide whether to give something at all and then decide, in a second step, how much to give. In a regression analysis, this two-stage decision process is most closely captured by a a two-part model (see Moffatt, 2016). To implement the two-part regression, we used a linear probability model (LPM) to model the binary decision to give any positive amount to the recipient in a first, and subsequently a gamma-GLM to assess how much a participant gave (conditional on giving a positive amount) in a second step. As the LPM results reported in the corresponding column of Table 3 indicate, compared to the NO DEFAULT treatment, the STRONG DEFAULT treatment significantly reduced the number of people who chose to give a positive amount to the recipient in Dictator Stage II. This negative effect is also significant when comparing the STRONG DEFAULT treatment to WEAK DEFAULT (*p* = 0.009), CONTROL INCOME (*p* = 0.024), and CONTROL PASSIVE GIVING (*p* = 0.021) using post-estimation F-tests. However, those participants in STRONG DEFAULT who did give something to the recipient, gave more than participants in NO DEFAULT, thus leading to the non-significantly different giving on average that we found in the OLS regression. Comparing the gamma-GLM coefficient of the dummy for the STRONG DEFAULT treatment to those of the two control conditions and to WEAK DEFAULT, we find that, conditional on giving a positive amount, there were no significant differences in giving across these conditions (*p* > 0.100 for all post-estimation Wald tests).

Thus, in sum, also the regression analyses confirm that, on average, neither the weak nor the strong default in our study led to negative spillover effects from initial giving choices on subsequent giving choices on average. The results from the two-part model provide some additional interesting insights, as the STRONG DEFAULT decreased the number of people willing to give anything in Dictator Stage II. However, this negative effect of the strong default on the propensity to give was compensated by higher giving by those participants who still decided to give something.

## 5. Discussion and Conclusions

In this study, we investigated the potential spillover effects of increased pro-social behavior triggered by pro-social choice defaults on not directly targeted, subsequent behavior. To do so, we contrasted subsequent pro-social behavior when there was no default, an easily changeable “weak” default, and a costly to switch “strong” default implemented to foster an initial pro-social behavior. We tested the potential spillover effects of behavior triggered by these choice defaults on subsequent behavior by applying a two-tiered control strategy taking into account potentially countervailing effects of different income levels and altruistic motivations stemming from the initial behavior.

Our findings provide important insights for policymakers and researchers alike. They carry good news for policymakers who make use of choice defaults for fostering pro-social choices, because both the non-obtrusive (weak) and the costly to switch (strong) default we implemented in our study did not cause problematic effects over time. Overall, the increase in pro-social giving triggered by the choice defaults did not lead participants to compensate and reduce their giving in a later choice without a default. Even though the STRONG DEFAULT led to fewer people making a positive transfer in Dictator Stage II, this effect was compensated by higher transfers by those participants who still decided to give something. Thus, while as intended—and in line with a large and growing literature documenting the effectiveness of choice defaults—the defaults we implemented in our study had a significant positive effect on the targeted pro-social behavior, there was no moral licensing in the form of negative spillover effects on subsequent behavior.

Our findings are further encouraging, because the increase in pro-social giving in Dictator Stage I triggered by the choice defaults was large, especially when considering the strong default treatment. The strong default more than doubled giving in Dictator Stage I compared to the no default condition and the effect size was large according to typical measures (Cohen's *d* = 0.74). These findings are important for researchers studying moral licensing. Given the existing literature on moral licensing, it is noteworthy that an intervention that increases pro-social behavior so strongly does not lead to any compensation in subsequent pro-social behavior. The absence of spillovers is even more notable given that the two behaviors were temporally very close to each other as they took place within a relatively short-lived laboratory session.

It could be argued that some features of our experimental design, specifically the filler task and the nature of the giving decision in Dictator Stage II, may have facilitated participants viewing the decisions as unrelated and thus favored the absence of spillovers. However, even though our observations and inferences are of course limited to the specific experimental set-up we implemented, we believe that this set-up provided an appropriate environment for detecting relevant spillover effects of pro-social behavior triggered by choice defaults on subsequent and similar pro-social decisions. First, the filler task lasted a maximum of 180 seconds during the conduct of the experiment. Hence, if it is the case that distractions, like filler tasks, are sufficient to eliminate potential spillover effects, it is unlikely that such spillovers are actually relevant in real-life decision making where the time that passes between potentially linked decisions is likely to be longer. Moreover, the use of filler tasks is common in studies following the sequential behavior paradigm, in order to ensure sufficient differentiation between initial and subsequent behavior (e.g., Sachdeva et al., 2009; Gneezy et al., 2012). Second, even though the recipient in the Dictator Game implemented in Dictator Stage II (another participant) was different than in Dictator Stage I (where it was a charity), conceptually the two decisions were highly similar. Both times the participants received a sum of money and decided how much to give to someone else. Previous studies have found negative or positive spillovers with behaviors that seem conceptually far more different than that, such as, for instance, saving water and electricity consumption (Tiefenbeck et al., 2013) or making a donation and telling the truth (Gneezy et al., 2012). Moreover, when designing the experiment we deliberately decided to implement a slightly different decision in Dictator Stage II compared to Dictator Stage I, as this case seems more relevant from a practical perspective. In reality, it is probably rarely the case that an individual faces the exact same pro-social decision again right away and that the first time it was subject to a choice default, whereas the second time it is not. Rather, and more relevantly from our perspective, the individual will likely face other pro-social decisions that are similar in the sense that they have a pro-social dimension to them, but that are not exactly the same. Thus, if behavioral spillovers matter for the overall effect of choice defaults on pro-social behavior, these spillover effects would need to be observed not on the exact same decision, but rather on related and similar—but not exactly identical—decisions.

Based on our data, we thus conclude that fostering pro-social decisions via the use of choice defaults—with or without significant costs to opt out—does not seem to influence non-targeted subsequent pro-social behavior. This is an encouraging finding for policy makers wanting to stimulate pro-social behavior via choice defaults, but fearing subsequent adverse effects.

Of course, our study is just a first step in the analysis of whether and how well-intended behavioral policy interventions such as choice defaults affect other, not directly targeted decisions and the potential spillover effects of choice defaults and other nudges should be investigated further in future research. One research question that should be explored in more detail is how spillover effects of such interventions depend on the nature of the subsequent behavior. As argued above, behavioral spillover effects seem to be of particular practical relevance if they occur not only on exactly identical subsequent decisions but also on related but not identical decisions. In general, it would be important to explore more systematically how this relatedness between behaviors affects spillover effects and what determines relatedness. Moreover, subsequent behavior may be due to and exposed to a large variety of contextual factors from which we abstracted in our laboratory study. Given the increasing popularity of nudging policies, it is important to increase our understanding about any desirable or undesirable side-effects such policy interventions may have. Especially, the evaluation of behavioral spillover effects of nudges in field-experimental settings would be important in this regard.

## Ethics Statement

This study was carried out in accordance with the recommendations of ETH Zurich's ethics commission with written informed consent from all subjects. All subjects gave written informed consent in accordance with the requirements of ETH Zurich's ethics commission. The protocol was approved by ETH Zurich's ethics commission.

## Author Contributions

CG, MG, and JS contributed equally to the conception of the study, the experimental design, and the final manuscript. CG managed the data collection, conducted the first data analyses and wrote the first version of the manuscript.

### Conflict of Interest Statement

The authors declare that the research was conducted in the absence of any commercial or financial relationships that could be construed as a potential conflict of interest.

## Footnotes

^1^Note that in this paper we narrow down the term spillover effects to the effect of an initial behavior triggered by the default on related *subsequent* behavior. In the literature, the term spillover effect is also used to describe the backfiring of policy instruments because of psychological reactance to a given policy leading to direct adverse effects on the targeted *initial* behavior (Schultz et al., 2007), or to explain so-called rebound effects due to individual adjustments to relative price changes, which are induced by a given policy (Alcott, 2005).

^2^These statements were taken from the website of Zewo Foundation, a Swiss institution that certifies charitable organizations with respect to integrity, efficient use of funds, and transparency, see www.zewo.ch/en/

^3^Appendix C in Supplementary Material displays the instructions provided to participants and screen-shots of the decision screens.

^4^What this condition does not control for is the warm-glow (Andreoni, 1990) stemming from the donation decision in Dictator Stage I. This is intentional, as it is exactly this warm-glow (i.e., the feeling of having done something good) which may yield a spillover effect and affect participants' decisions in Dictator Stage II (Schmitz, forthcoming).

^5^One treatment session was conducted in a within fashion due to unbalanced show up of participants. Results of this single session are not significantly different with respect to the remaining sessions [Kolmogorov-Smirnov test *n*_1_ = 24, *n*_2_ = 234, *p* = 0.435 (distribution of giving in Dictator Stage I), *n*_1_ = 24, *n*_2_ = 234, *p* = 0.139 (distribution of giving in Dictator Stage II)].

^6^The complete data-set and the *R* code for all analyses reported in the paper can be downloaded from https://figshare.com/s/a5ed8c829c7c0c80e2f5

^7^Although, we have directed hypotheses, we rely on two-sided tests for all inferential testing in this paper. We use t-tests to test for statistical significance of differences in giving. Results from non-parametric Wilcoxon rank-sum tests yield highly similar results and are available on request.

^8^For the *t*-tests reported in this section, we only considered the exact matches of income for each treatment condition in order to ensure perfect comparability. In the regressions reported in Table 3 we use the full data from the control conditions when controlling for possible income effects and can thus increase statistical power.

^9^In this vein, note that the main effect coefficients for “Income before DG II” in the regressions reported in Table 3 do not capture a causal income effect. Because the regressions also contain the interaction terms of this variable with the dummies for the experimental conditions, the coefficients for “Income before DG II” apply to the NO DEFAULT treatment, in which the “income” (i.e., the money a participant had earned in the experiment before entering Dictator Stage II) was determined by the participant's own donation decision in Dictator Stage I. Thus, the negative coefficients we find in the regressions are due to self-selection (as participants with a tendency to give little in Dictator Stage I also give little in Dictator Stage II). A causal income effect can be estimated in the CONTROL INCOME condition and corresponds to testing that the sum of the coefficients for “Income before DG II” and the interaction term “CONTROL INCOME x Income before DG II” is different from zero. We do not find evidence for a significant income effect on average giving in Dictator Stage II (*p* = 0.967, post-estimation F-test based on OLS results). The corresponding test for the CONTROL PASSIVE GIVING condition reveals that there is also no significant income effect when adding altruistic utility (*p* = 0.938, post-estimation F-test).
