# Supplementary material for: Nudge for Good? Choice Defaults and Spillover Effects
Source: Front Psychol. 2019 Feb 12;10:178. doi: 10.3389/fpsyg.2019.00178 (PMC6379727; doi:10.3389/fpsyg.2019.00178)
Supplement: Supplementary file 1 [file Presentation_1.pdf]

APPENDIX to:

Nudge for Good? Choice Defaults and Spillover Effects

Claus Ghesla* Manuel Grieder† Jan Schmitz†

January 31, 2019

*Institute for Advanced Studies (IHS), Insight Austria, Vienna, Austria, ghesla@ihs.ac.at

†Swiss Federal Institute of Technology (ETH) Zurich; Department of Humanities, Social and Political Sciences; Chair of Economics; Zurich, Switzerland, manuel.grieder@econ.gess.ethz.ch, jan.schmitz@econ.gess.ethz.ch

Appendix

A Sample characteristics

Table A1: Sample characteristics

	NO DEFAULT	WEAK DEFAULT	STRONG DEFAULT	CONTROL INCOME	CONTROL PASSIVE GIVING	Overall
N	129	129	128	148	144	678
Age	23.1	24.0	22.2	22.8	22.6	22.9
Gender=F	56%	56%	50%	55%	47%	53%
Income	84%	82%	81%	85%	88%	84%
Education	92%	95%	98%	96%	95%	95%
Extraversion	3.25	3.06	3.19	3.17	3.20	3.17
Agreeableness	4.12	4.09	4.12	4.16	3.96	4.08
Conscientiousness	3.56	3.59	3.64	3.65	3.69	3.61
Neuroticism	2.83	2.57	2.63	2.79	2.69	2.72
Intellect	3.73	3.88	3.84	3.86	3.71	3.81
Need for Cognition	3.58	3.66	3.62	3.54	3.43	3.59
Reactance	3.04	2.89	2.91	2.86	2.89	2.93
Regret	3.37	3.21	3.16	3.33	3.35	3.29
IQ	8.23	8.37	8.24	8.57	8.32	8.31

Note.— The table shows sample characteristics for each of the five experimental conditions and additionally provides overall statistics. Age is reported as a mean. Gender indicates the proportion of female participants. Income denotes the share of participants with a monthly income below CHF 2,000. Education denotes the share of participants with A-levels or higher. Extraversion, Agreeableness, Conscientiousness, Neuroticism, and Intellect were measured on a 5-point Likert-scale using the mini-IPIP scales (Donnellan et al., 2006). Need for Cognition (Beissert et al., 2014), reactance (Herzberg, 2002), and regret (Schwartz et al., 2002) were also measured on 5-point Likert scale. IQ was measured with 12 items from the IQ-test by Catell (1940). Scores are denoted as means. Contingency tests performed for the complete sample show no significant differences in participant characteristics across treatment and control conditions.

B Income matching procedure for control conditions

We applied the same income matching to both control conditions, CONTROL INCOME and CONTROL PASSIVE GIVING. In these two control conditions, participants received an additional income on top of their participation fee that matched the monetary impact of the choices of participants in NO DEFAULT, WEAK DEFAULT and STRONG DEFAULT. For instance, if a participant in WEAK DEFAULT decided to donate 10 points to a charity, then the remaining 'income' that this participant took into Dictator Stage II was 90 points. In this case, a matched participant in CONTROL INCOME (respectively CONTROL PASSIVE GIVING) received an additional 90 points on top of his / her show-up fee. In this example, the participant from WEAK DEFAULT and the respective control participants thus arrived at Dictator Stage II with the exact same amount of money earned in the experiment up to that point. We did not conduct an exact one-to-one matching, as we had more participants in the NO DEFAULT, WEAK DEFAULT and STRONG DEFAULT treatments than in the control conditions. Our matching procedure ensured, however, that the distributions of 'Income before DG II' were identical in the treatment and the matched control condition. Table A2 shows test statistics for the two-sample Kolmogorov-Smirnov-tests for differences in these distributions. The null hypothesis is that the distributions are equal and that the test statistic D is not statistically different from zero.

Table A2: Kolmogorov-Smirnov test statistics

Distribution Comparison	n_1	n_2	D	p-value
NO DEFAULT | CONTROL INCOME	129	49	0.044	1.000
NO DEFAULT | CONTROL PASSIVE GIVING	129	46	0.031	1.000
WEAK DEFAULT | CONTROL INCOME	129	49	0.024	1.000
WEAK DEFAULT | CONTROL PASSIVE GIVING	129	46	0.033	1.000
STRONG DEFAULT | CONTROL INCOME	128	50	0.026	1.000
STRONG DEFAULT | CONTROL PASSIVE GIVING	128	52	0.022	1.000

Note.— Test statistics (D) and p-value for Kolmogorov-Smirnov tests for comparison of income distribution equality among treatment and control conditions. Low values of D suggest that distributions of income do not differ between the corresponding groups. P-values below conventional levels would lead to the rejection of the hypothesis that the underlying distributions are equal.

C Experimental Instructions

Note: This set of translated instructions was used for respondents in NO DEFAULT, WEAK DEFAULT and STRONG DEFAULT. Differences in WEAK DEFAULT and respectively STRONG DEFAULT are *italicized*. In CONTROL INCOME, Dictator Stage I was omitted and participants solely received information about their endowment (= participation fee). The remainder of the experimental instructions was identical to NO DEFAULT / WEAK DEFAULT / STRONG DEFAULT. In CONTROL PASSIVE GIVING participants again received information about their endowment (= participation fee). Subsequently, they were presented with the same instructions as in Dictator Stage I. However, they were told that independent of their income an amount between 0 and 100 points would be donated to a charity of their choice.[1] Hence, they could read all the information about the charities and pick one to which the money was donated. Participants were also able to let the computer decide randomly on the choice of a charity. The amount of the donations could not be influenced by the participants. Subsequent to their decision of choosing a charity, they received feedback about the amount of points that was donated. Afterwards the instructions were identical to those in NO DEFAULT/ WEAK DEFAULT / STRONG DEFAULT.

General Explanations for Participants

Welcome to the experimental laboratory. Today you are taking part in a scientific study, in which you can earn a certain amount of money, which will be handed to you in cash. How much money you earn, is dependent on your decisions and the decisions of other participants. Therefore, please read these instructions carefully.

The set of instructions is for your private use only. Please do not communicate with other participants during the experiment. If you have questions, give a hand signal and the experimenter will come to your desk to answer your questions. Non-observance of this rule will lead to the exclusion of the experiment. During the experiment you will receive information on your computer screen.[2] You take your decisions with keyboard and mouse. Your inputs are completely anonymous. The experimenter knows your identity, however we are not able to relate your decisions with your identity.

Please only use the buttons within the experimental window. With the button 'Continue' and respectively 'Back' you are able to change between the next and the previous page (if possible).

This study consists of **five parts**, in which you receive information and need to make decisions, which may influence your payoff. Your payoff will be calculated in points and converted according to the following rule:

10 Points = 1 Swiss Franc

[1]This procedure follows the instructions by Gneezy et al. (2012) for a 'costless' donation.

[2]Note that these instructions are supplemented with figures from the actual program, as we did not use paper instructions.

How much you can earn in each of the parts will be stated in the instructions, which will be shown for each part separately on the screen. At the end of the study, the points you have earned will be converted to Swiss Francs and paid out in cash to you.

The study ends with a short questionnaire. As soon as each participant has completed this questionnaire, the pay-out will be started. You will be called for pay-out by your seat number. Expected processing time for the study is between 45 to 60 minutes.

Dictator Stage I - NO DEFAULT/WEAK DEFAULT

In this part of the study you receive **200 points**. 100 points thereof are your participation premium, which you can keep with certainty. The other 100 points are at your disposal for your decision in this part of the study. You can thereby decide how to allocate these 100 points (in increments of 10 points) between yourself and a charity. You can keep all points for yourself and give no points to a charity; you can devote all points to a charity and keep no points for yourself; or you can keep a certain amount of points for yourself and pass the remaining points to a charity. The amount of your donation can be specified with in the input field 'Ihre Spende [in Punkten]' (Your Donation [in Points]).[3]

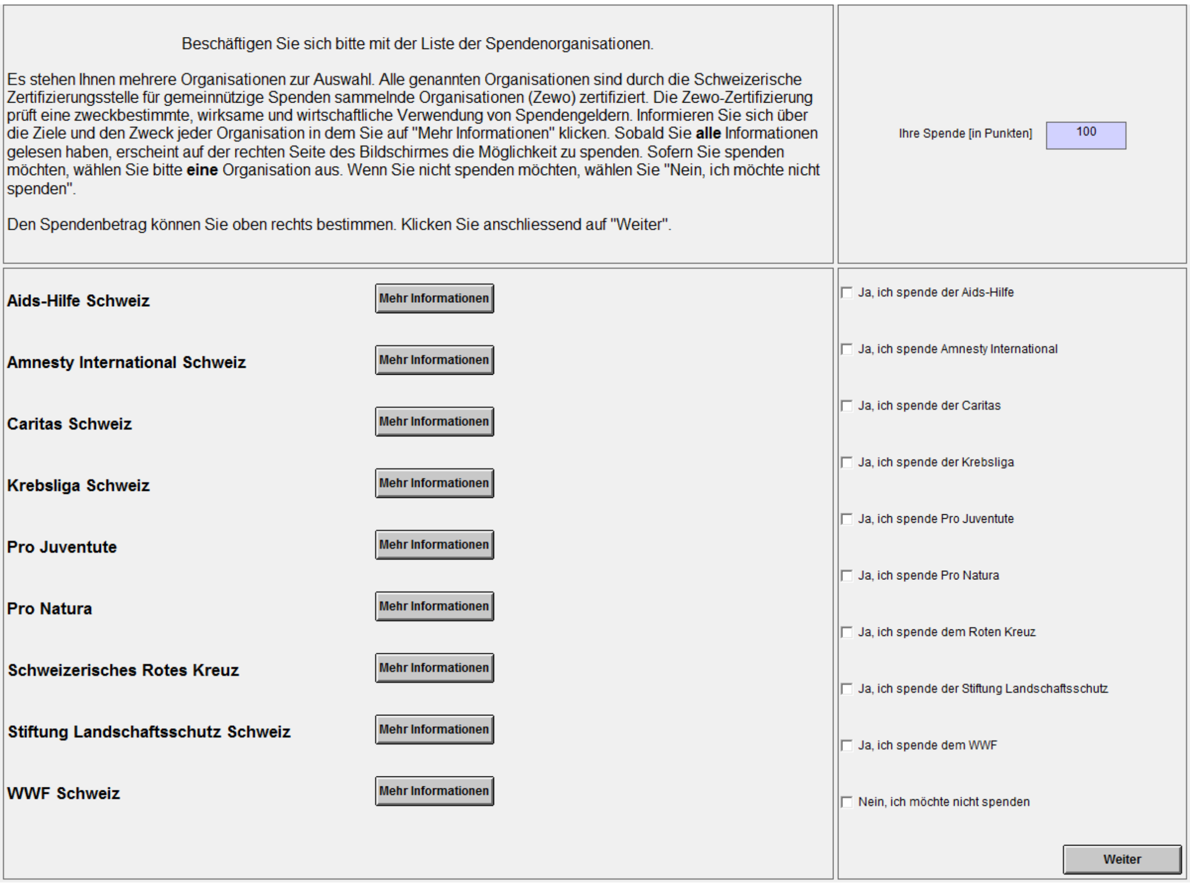

Figure A1: Sample screen of a decision task in the Dictator Stage I in Experiment I.
Note.— WEAK DEFAULT is simply implemented by pre-specifying the input field to '100'. In NO DEFAULT this field initially remains blank.

There are **nine charities** available for selection, which will be described on the left-hand side of the screen. All charities are certified by the 'Swiss Zewo Foundation'. The 'Zewo Foundation' testifies a purposive, effective and economic use of donation money. Inform yourself on the goals and purpose of each charity by clicking on 'Mehr Informationen' (More Information). The button 'Mehr Informationen' changes its color from red to grey, once you have read the information about a charity.

[3] See Figure A1 for a screen of the decision.

As soon as you have read the **complete** set of information about each charity, you will be able to select a charity to donate to on the right hand side of the screen. In case that you want to allocate points between yourself and a charity, please select **one** charity. You can only choose one charity to donate to. *With clicking on "Weiter" (Continue) you donate 100 points to a selected charity. You can specify a different donation amount in the upper right corner (WEAK DEFAULT).* If you do not want to allocate points between yourself and a charity, please choose "Nein, ich möchte nicht spenden." (No, I do not want to donate.)

Please note that points, which you keep for yourself, will be paid out in cash at the end of the study. Points, which you allocate to a charity will be donated by the experimenter to the chosen charity. If you donate, you will receive an official letter by the Chair of Economics at ETH Zurich with your pay-out that the chosen amount will be transfered to the corresponding charity. In order to familiarize yourself with the decision task, please answer the following questions: Person A donates 40 points to a charity.

(1) How many points will person A receive at the end of the study with this decision? (Please note that you will keep 100 of your 200 points with certainty.)
(2) How many points will the charity receive at the end of the study with this decision?

Dictator Stage I - STRONG DEFAULT

In this part of the study you receive **200 points**. 100 points thereof are your participation premium, which you can keep with certainty. The other 100 points are at your disposal for your decision in this part of the study. You can thereby decide how to allocate these 100 points (in increments of 10 points) between yourself and a charity. You can keep all points for yourself and give no points to a charity; you can devote all points to a charity and keep no points for yourself; or you can keep a certain amount of points for yourself and pass the remaining points to a charity. The amount of your donation can be specified with in the input field 'Ihre Spende [in Punkten]' (Your Donation [in Points]).[4]

There are **nine charities** available for selection, which will be described on the left-hand side of the screen. All charities are certified by the 'Swiss Zewo Foundation'. The 'Zewo Foundation' testifies a purposive, effective and economic use of donation money. Inform yourself on the goals and purpose of each charity by clicking on 'Mehr Informationen' (More Information). The button 'Mehr Informationen' changes its color from red to grey, once you have read the information about a charity.

As soon as you have read the **complete** set of information about each charity, you will be able to select a charity to donate to on the right hand side of the screen. In case that you want to allocate points between yourself and a charity, please select **one** charity. You can only choose one charity to donate to. With clicking on "Weiter" (Continue) you donate 100 points to a selected charity. You can specify a different donation amount in the upper right corner. If you do not want to allocate points between yourself and a charity, please choose "Nein, ich möchte nicht spenden." (No, I do not want to donate.)

[4]see Figure A1 for a decision screen

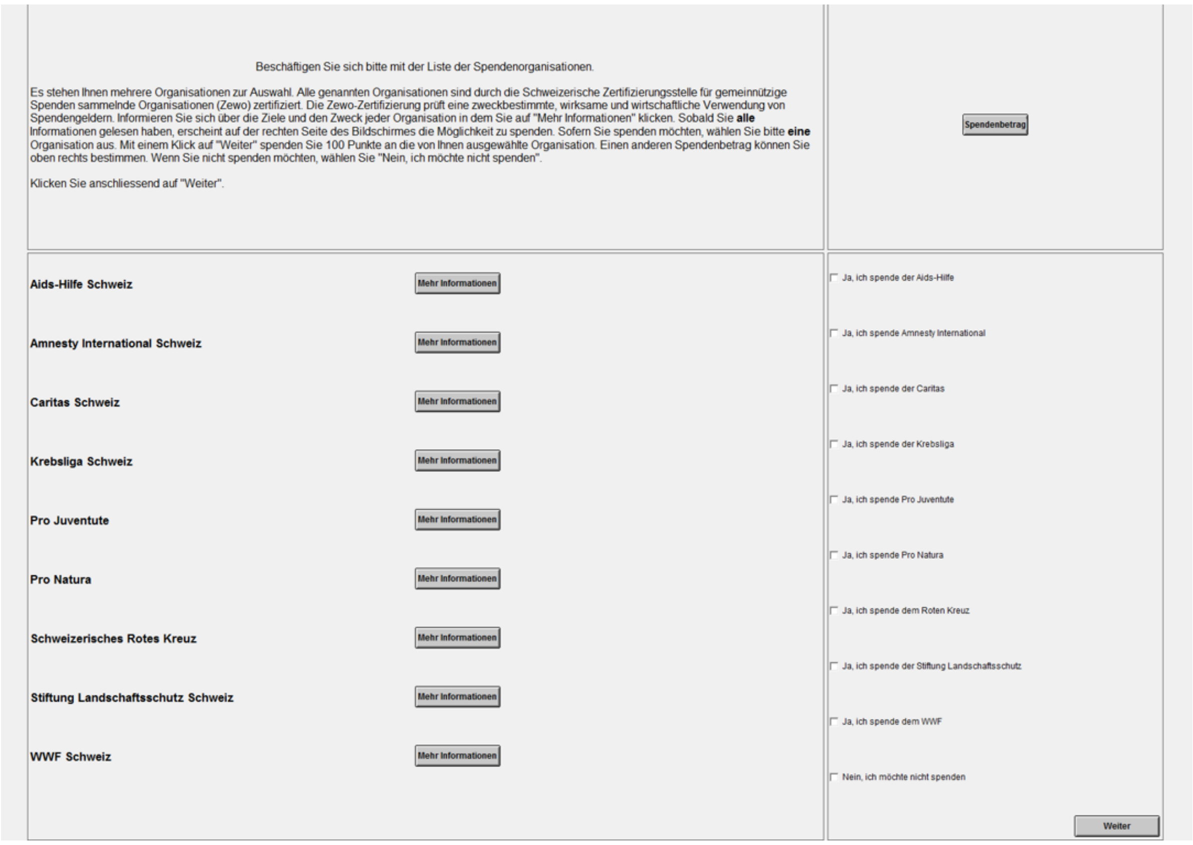

Figure A2: Sample screen of the decision task in the Dictator Stage I in STRONG DEFAULT.

Once the button in the upper right corner is pressed, participants receive additional information on how to change the donation amount (STRONG DEFAULT):
To change your donation of 100 points, you need to fulfill a task. The task consists of changing 48 sliders with your mouse. Each slider is initially positioned at 0 and can be moved as far as 100. A number right to the slider indicates its current position. You can readjust the position of each slider as many times as you wish. You have to adjust all sliders to the value 50 - only then you will be able to change the donation amount. If you do not like to fulfill the task, please click on ‘Abbrechen’ (Cancel).

Please note that points, which you keep for yourself, will be paid out in cash at the end of the study. Points, which you allocate to a charity will be donated by the experimenter to the chosen charity. If you donate, you will receive an official letter by the Chair of Economics at ETH Zurich with your pay-out that the chosen amount will be transfered to the corresponding charity. In order to familiarize yourself with the decision task, please answer the following questions: Person A donates 40 points to a charity.

(1) How many points will person A receive at the end of the study with this decision? (Please note that you will keep 100 of your 200 points with certainty.)
(2) How many points will the charity receive at the end of the study with this decision?

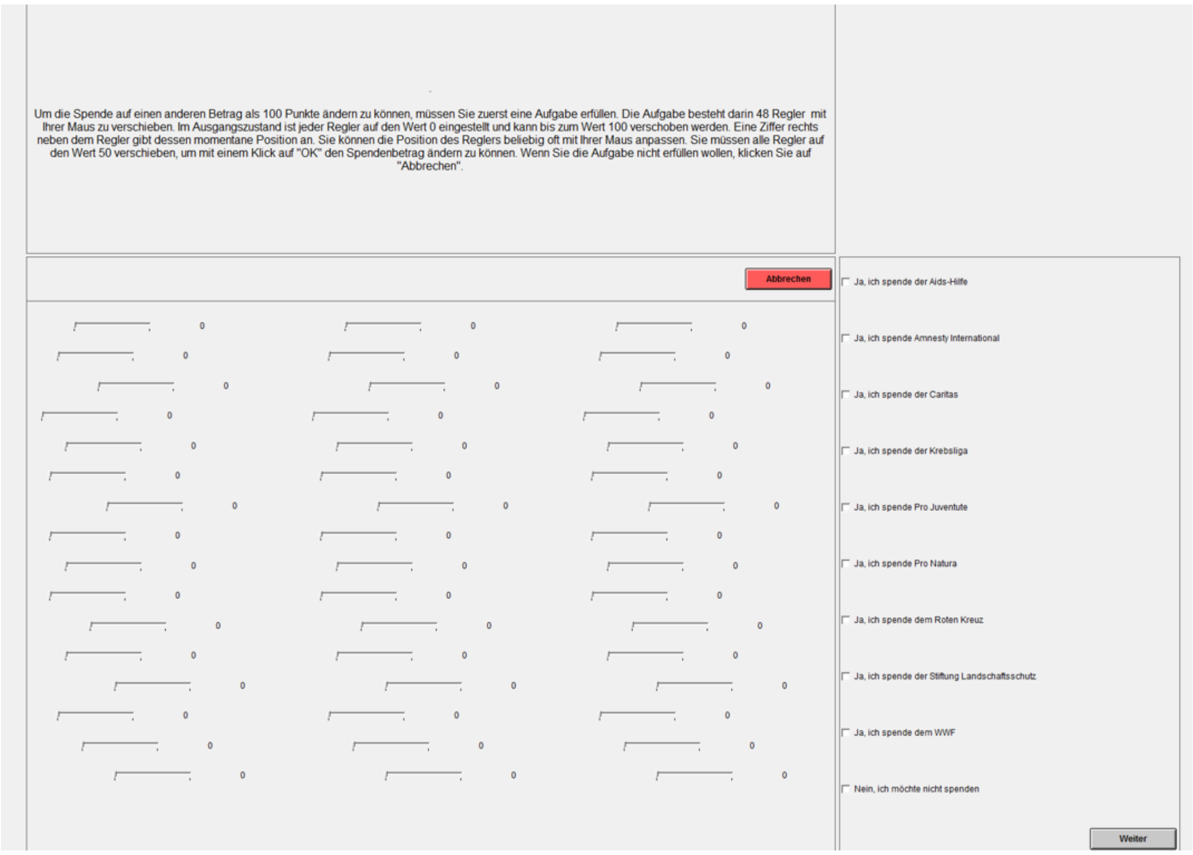

Figure A3: Sample screen of the slider task in the Dictator Stage I in STRONG DEFAULT.

Filler task: Shortened IQ-test after Catell (1940)

Note: The IQ-test was divided into two parts, which share exactly the same instructions. In each part, participants had to identify a subset of four figures. Exemplarily, we show a figure of each subset.

Section 2 consists of a shortened version of an intelligence test. The tests is divided into two parts. For each part you receive further information.

The figure shown below (see Figure A4 and A5 for an example in each part) gives you an example of the exercise you have to solve in part one (or two). You have to decide which of the squares on the right hand side follows logically the squares on the left (fits logically into the larger square on the left). You make your choice by clicking on the button below the squares. In this example you should choose ‘c’ (‘b’), because the circles in the squares get smaller from square to square (because it fits exactly with the smaller upper right square).

The test starts as soon as you click the button ‘Start’. You have 1 minute and 30 seconds to answer each part. Probably, the amount of time allowed is not sufficient to answer all questions. Do not let yourself discourage by this. Simply work as correctly and as fast as possible.

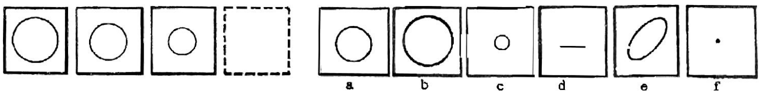

Figure A4: Sample exercise in part one of the IQ-test

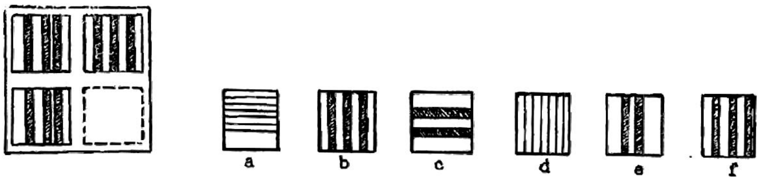

Figure A5: Sample exercise in part two of the IQ-test

Dictator Stage II

In this section of the study you have to decide on the distribution of **200 points** between yourself and a randomly allocated person in this room. This allocation is anonymous. Neither you nor the other person gets to know your mutual identities during or after the study. You decide in anonymity. Your own decision can not be influenced by the other person.

You have to decide how many points you want to give to the other person. You have 200 points for your decisions. You can keep all points for yourself and give no points to the other person; you can give all points to the other person and keep no points for yourself; or you can keep a certain amount of points (in increments of 10 points) and pass the remaining points to the other person. You can specify the number of points you want to give to the other person in the designated input field.

The other, randomly allocated, person has the same decision task as you and needs to decide how many of the 200 points she or he wants to give to you. **However, only one of these two decisions will be implemented, i.e., the 200 points will be distributed among you and the other person only once.** Which of these two decisions is relevant will be determined randomly by the computer. If the computer (with a probability of 0.5) randomly determines that your decision will be implemented, the other participant will receive the points that you have decided to give to her or him. If the computer (with a probability of 0.5) randomly determines that the decision of the other participant is implemented, you will receive the points that the other participant has decided to give to you. As you are unable to determine whether the computer selects your or the other person's decision, you should carefully consider the decision task.

If your decision is implemented, you will receive the points, which you have kept for yourself and these points will be paid out in cash at the end of the study. If the decision of the other person is implemented, you will receive the points that the other person has given to you, and the other person keeps the remaining points.

In order to familiarize yourself with the decision task, please answer the following questions: Person A gives 70 points to person B. Person B gives 10 points to person A. The computer implements the decision of person B.

(1) How many points will person A receive at the end of the study with this decision?
(2) How many points will person B receive at the end of the study with this decision?

References

Beissert, H., Köhler, M., Rempel, M., & Beierlein, C. (2014). Eine deutschsprachige Kurzskala zur Messung des Konstrukts Need for Cognition. Die Need for Cognition Kurzskala (NFC-K). Working Paper 32, Leibniz-Institut für Sozialwissenschaft.

Catell, R. B. (1940). A culture-free intelligence test. *Journal of Educational Psychology*, *31*(3), 161–179.

Donnellan, B. M., Oswald, F. L., Baird, B. M., & Lucas, R. E. (2006). The mini-IPIP scales: Tiny-yet-effective measures of the Big Five factors of personality. *Psychological Assessment*, *18*(2), 192–203.

Gneezy, A., Imas, A., Leif, N. D., Brown, A., & Norton, M. I. (2012). Paying to be Nice: Consistency and Costly Procsocial Behavior. *Management Science*, *58*(1), 179–187.

Herzberg, P. Y. (2002). Zur psychometrischen Optimierung einer Reaktanzskala mittels klassischer und IRT-basierter Analysemethoden. *Diagnostica*, *48*, 163–171.

Schwartz, B., Ward, A., Monterosso, J., Lyubomirsky, S., White, K., & Lehman, D. R. (2002). Maximizing versus satisficing: Happiness is a matter of choice. *Journal of Personality and Social Psychology*, *83*, 1178–1197.
